# Bis{4-chloro-6-formyl-2-[(*E*)-2-(1*H*-imidazol-4-yl-κ*N* ^3^)ethyl­imino­methyl-κ*N*]phenolato-κ*O* ^1^}nickel(II)

**DOI:** 10.1107/S1600536808031577

**Published:** 2008-10-04

**Authors:** Jia-Wei Mao, Hong Zhou, Zhi-Quan Pan, Xiang-Gao Meng

**Affiliations:** a Key Laboratory for Green Chemical Process of Ministry of Education, Wuhan Institute of Technology, Wuhan 430073, People’s Republic of China; b Department of Chemistry, Central China Normal University, Wuhan 430079, People’s Republic of China

## Abstract

In the title compound, [Ni(C_13_H_11_ClN_3_O_2_)_2_], the Ni^II^ atom is located on a twofold rotation axis and is six-coordinated by four N atoms and two phenolate O atoms from the two equal Schiff base ligands in a distorted octa­hedral coordination geometry. The complex mol­ecules are connected by C—H⋯Cl, C—H⋯O and N—H⋯O hydrogen bonds.

## Related literature

For related literature on transition metal–Schiff base complexes, see: Casella & Gullotti (1986 ▶); Hodnett & Dunn (1970 ▶); Kim *et al.* (2005 ▶); May *et al.* (2004 ▶). For literature related to the synthesis, see: Taniguchi (1984 ▶).
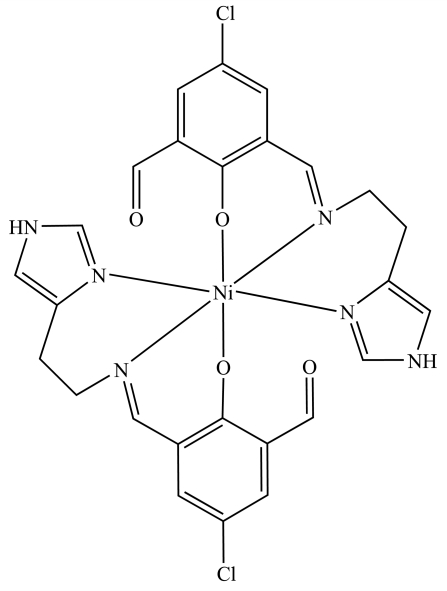


## Experimental

### 

#### Crystal data

[Ni(C_13_H_11_ClN_3_O_2_)_2_]
*M* *_r_* = 612.11
Tetragonal, 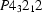

*a* = 13.5883 (16) Å
*c* = 14.0392 (16) Å
*V* = 2592.2 (5) Å^3^
*Z* = 4
Mo *K*α radiation
μ = 1.00 mm^−1^
*T* = 293 (2) K
0.10 × 0.04 × 0.02 mm

#### Data collection

Bruker SMART APEX CCD area-detector diffractometer
Absorption correction: multi-scan (*SADABS*; Sheldrick, 1996 ▶) *T*_min_ = 0.901, *T*_max_ = 0.978
21136 measured reflections
2294 independent reflections
1253 reflections with *I* > 2σ(*I*)
*R*_int_ = 0.154

#### Refinement

*R*[*F* ^2^ > 2σ(*F* ^2^)] = 0.045
*wR*(*F* ^2^) = 0.081
*S* = 0.82
2294 reflections
177 parameters
H-atom parameters constrained
Δρ_max_ = 0.35 e Å^−3^
Δρ_min_ = −0.24 e Å^−3^
Absolute structure: Flack (1983 ▶), 920 Friedel pairs
Flack parameter: 0.02 (3)

### 

Data collection: *SMART* (Bruker, 2007 ▶); cell refinement: *SAINT* (Bruker, 2007 ▶); data reduction: *SAINT*; program(s) used to solve structure: *SHELXS97* (Sheldrick, 2008 ▶); program(s) used to refine structure: *SHELXL97* (Sheldrick, 2008 ▶); molecular graphics: *SHELXTL* (Sheldrick, 2008 ▶); software used to prepare material for publication: *SHELXTL*.

## Supplementary Material

Crystal structure: contains datablocks global, I. DOI: 10.1107/S1600536808031577/hy2154sup1.cif

Structure factors: contains datablocks I. DOI: 10.1107/S1600536808031577/hy2154Isup2.hkl

Additional supplementary materials:  crystallographic information; 3D view; checkCIF report

## Figures and Tables

**Table:** 

Ni1—O1	2.054 (3)
Ni1—N2	2.068 (4)
Ni1—N1	2.102 (4)

**Table:** 

O1—Ni1—O1^i^	87.37 (17)
O1—Ni1—N2^i^	91.40 (14)
O1—Ni1—N2	178.49 (14)
N2^i^—Ni1—N2	89.8 (2)
O1—Ni1—N1	88.84 (14)
N2—Ni1—N1	90.27 (15)
O1—Ni1—N1^i^	89.72 (13)
N2—Ni1—N1^i^	91.14 (15)
N1—Ni1—N1^i^	178.0 (2)

Symmetry code: (i) 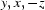.

**Table 2:** Hydrogen-bond geometry (Å, °)

*D*—H⋯*A*	*D*—H	H⋯*A*	*D*⋯*A*	*D*—H⋯*A*
C9—H9*B*⋯Cl1^ii^	0.97	2.82	3.475 (5)	125
C12—H12⋯O2^iii^	0.93	2.36	3.287 (7)	174
N3—H3*A*⋯O1^iv^	0.86	2.06	2.899 (5)	166

Symmetry codes: (ii) 
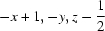
; (iii) 
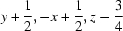
; (iv) 
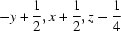
.

**Fig. 1.**
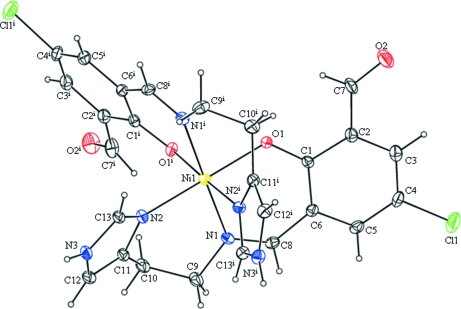
Molecular structure of the title compound. Displacement ellipsoids are drawn at the 30% probability level. [Symmetry code: (i) x, y, -z.]

## supplementary crystallographic information

### Comment

Transition metal–Schiff base complexes have been an interesting field for a long time due to their striking biological activites (Casella & Gullotti, 1986; Hodnett & Dunn, 1970; Kim *et al.*, 2005; May *et al.*, 2004). In this paper, we report the crystal structure of a new nickel(II) complex with a Schiff base ligand, 2-[(*E*)-(2-(1*H*-imidazol-4-yl)ethylimino)methyl]-4-chloro -6-formylphenolate.

In the title compound, the Ni^II^ atom is located on a twofold rotation axis and six-coordinated by four N atoms and two phenolate O atoms from two Schiff base ligands (Fig. 1). The coordination geometry of the Ni atom can be described as distorted octahedral. The two phenolate O atoms and the two imidazole N atoms are located in the equatorial plane, with Ni—O distance of 2.054 (3)Å and Ni—N distance of 2.068 (4)Å (Table 1), and with the mean plane deviation of 0.0147 (2) Å. The other two N atoms from the imino groups of the Schiff base ligands occupy the axial positions, with somewhat long Ni—N distance of 2.102 (4) Å. The complex molecules are connected by C—H···Cl, C—H···O and N—H···O hydrogen bonds (Table 2).

### Experimental

2,6-Diformyl-4-chlorophenol was prepared using the method of Taniguchi (1984). The title compound was synthesized by the following procedure: To an acetonitrile solution (10 ml) of 2,6-diformyl-4-chlorophenol (0.092 g, 0.5 mmol) and Ni(ClO_4_)_2_.6H_2_O (0.018 g, 0.25 mmol), a solution of NaOH (0.041 g, 1 mmol) and histamine dihydrochloride (0.092 g, 0.5 mmol) in 15 ml of absolute methanol was added dropwise. After the mixture was stirred at ambient temperature for about 1 h, a red solution appeared and then the stirring was continued for 3 h. Red needle crystals of the title compound suitable for X-ray diffraction were obtained in about a month.

### Refinement

H atoms were positioned geometrically and refined as riding atoms, with C—H = 0.93(CH), 0.97(CH_2_) Å and N—H = 0.86 Å and with *U*_iso_(H) = 1.2*U*_eq_(C,*N*).

### Crystal data

**Table:**

[Ni(C_13_H_11_ClN_3_O_2_)_2_]	*D*_x_ = 1.568 Mg m^−^^3^
*M**_r_* = 612.11	Mo *K*α radiation, λ = 0.71073 Å
Tetragonal, *P*4_3_2_1_2	Cell parameters from 1360 reflections
Hall symbol: P 4nw 2abw	θ = 2.6–15.1°
*a* = 13.5883 (16) Å	µ = 1.00 mm^−^^1^
*c* = 14.0392 (16) Å	*T* = 293 K
*V* = 2592.2 (5) Å^3^	Needle, red
*Z* = 4	0.10 × 0.04 × 0.02 mm
*F*(000) = 1256	

### Data collection

**Table:**

Bruker SMART APEX CCD area-detector diffractometer	2294 independent reflections
Radiation source: fine-focus sealed tube	1253 reflections with *I* > 2σ(*I*)
graphite	*R*_int_ = 0.154
φ and ω scans	θ_max_ = 25.0°, θ_min_ = 2.1°
Absorption correction: multi-scan (SADABS; Sheldrick, 1996)	*h* = −16→16
*T*_min_ = 0.901, *T*_max_ = 0.978	*k* = −16→16
21136 measured reflections	*l* = −14→16

### Refinement

**Table:**

Refinement on *F*^2^	Secondary atom site location: difference Fourier map
Least-squares matrix: full	Hydrogen site location: inferred from neighbouring sites
*R*[*F*^2^ > 2σ(*F*^2^)] = 0.045	H-atom parameters constrained
*wR*(*F*^2^) = 0.081	*w* = 1/[σ^2^(*F*_o_^2^) + (0.0298*P*)^2^] where *P* = (*F*_o_^2^ + 2*F*_c_^2^)/3
*S* = 0.82	(Δ/σ)_max_ = 0.003
2294 reflections	Δρ_max_ = 0.35 e Å^−^^3^
177 parameters	Δρ_min_ = −0.24 e Å^−^^3^
0 restraints	Absolute structure: Flack (1983), 920 Friedel pairs
Primary atom site location: structure-invariant direct methods	Flack parameter: 0.02 (3)

### Special details

**Table:**

Geometry. All e.s.d.'s (except the e.s.d. in the dihedral angle between two l.s. planes) are estimated using the full covariance matrix. The cell e.s.d.'s are taken into account individually in the estimation of e.s.d.'s in distances, angles and torsion angles; correlations between e.s.d.'s in cell parameters are only used when they are defined by crystal symmetry. An approximate (isotropic) treatment of cell e.s.d.'s is used for estimating e.s.d.'s involving l.s. planes.
Refinement. The reason of the large *R*_int_value is the poor quality and small size of the crystal sample. Although many efforts were made to select better crystal for experiment, each time we failed.

### Fractional atomic coordinates and isotropic or equivalent isotropic displacement parameters (Å^2^)

**Table:**

	*x*	*y*	*z*	*U*_iso_*/*U*_eq_	
Ni1	0.23545 (4)	0.23545 (4)	0.0000	0.0433 (3)	
C1	0.2375 (4)	0.0575 (3)	0.1277 (3)	0.0428 (12)	
C2	0.2104 (4)	0.0113 (4)	0.2139 (4)	0.0564 (15)	
C3	0.2586 (4)	−0.0728 (4)	0.2478 (4)	0.0656 (14)	
H3	0.2381	−0.1026	0.3041	0.079*	
C4	0.3356 (4)	−0.1106 (4)	0.1977 (5)	0.0642 (17)	
C5	0.3666 (4)	−0.0659 (4)	0.1150 (4)	0.0593 (15)	
H5	0.4202	−0.0920	0.0825	0.071*	
C6	0.3205 (4)	0.0165 (3)	0.0790 (4)	0.0455 (13)	
C7	0.1300 (4)	0.0521 (5)	0.2722 (4)	0.0800 (19)	
H7	0.1037	0.1125	0.2545	0.096*	
C8	0.3581 (3)	0.0522 (4)	−0.0096 (4)	0.0519 (13)	
H8	0.4055	0.0129	−0.0384	0.062*	
C9	0.3904 (4)	0.1472 (4)	−0.1444 (4)	0.0700 (17)	
H9A	0.4022	0.0839	−0.1743	0.084*	
H9B	0.4539	0.1760	−0.1294	0.084*	
C10	0.3368 (4)	0.2136 (4)	−0.2143 (3)	0.0636 (15)	
H10A	0.3701	0.2112	−0.2754	0.076*	
H10B	0.2704	0.1892	−0.2233	0.076*	
C11	0.3326 (4)	0.3166 (4)	−0.1810 (4)	0.0500 (14)	
C12	0.3728 (4)	0.4001 (4)	−0.2169 (4)	0.0620 (16)	
H12	0.4097	0.4056	−0.2724	0.074*	
C13	0.2960 (3)	0.4340 (4)	−0.0856 (4)	0.0504 (14)	
H13	0.2707	0.4695	−0.0345	0.060*	
Cl1	0.39581 (12)	−0.21688 (11)	0.23773 (13)	0.1081 (7)	
N1	0.3352 (3)	0.1319 (3)	−0.0552 (3)	0.0487 (11)	
N2	0.2844 (3)	0.3389 (3)	−0.0971 (3)	0.0480 (11)	
N3	0.3486 (3)	0.4739 (3)	−0.1563 (3)	0.0584 (12)	
H3A	0.3641	0.5349	−0.1622	0.070*	
O1	0.1862 (2)	0.1302 (2)	0.0935 (2)	0.0494 (9)	
O2	0.0962 (3)	0.0119 (3)	0.3416 (3)	0.1061 (15)	

### Atomic displacement parameters (Å^2^)

**Table:**

	*U*^11^	*U*^22^	*U*^33^	*U*^12^	*U*^13^	*U*^23^
Ni1	0.0453 (3)	0.0453 (3)	0.0393 (5)	0.0010 (4)	0.0040 (3)	−0.0040 (3)
C1	0.038 (3)	0.050 (3)	0.040 (3)	−0.012 (3)	−0.004 (3)	0.003 (3)
C2	0.057 (4)	0.056 (4)	0.056 (4)	−0.014 (3)	−0.008 (3)	0.009 (3)
C3	0.067 (4)	0.064 (4)	0.066 (4)	−0.024 (3)	−0.020 (5)	0.016 (4)
C4	0.059 (4)	0.055 (4)	0.079 (5)	0.001 (3)	−0.026 (4)	0.017 (4)
C5	0.041 (3)	0.060 (4)	0.077 (5)	−0.003 (3)	−0.011 (3)	−0.002 (3)
C6	0.043 (3)	0.043 (3)	0.050 (4)	−0.003 (3)	−0.006 (3)	0.001 (3)
C7	0.080 (5)	0.115 (5)	0.045 (5)	−0.021 (4)	0.001 (4)	0.030 (4)
C8	0.048 (3)	0.044 (3)	0.064 (4)	0.007 (3)	0.004 (3)	−0.013 (3)
C9	0.094 (4)	0.056 (4)	0.060 (4)	0.015 (3)	0.028 (4)	0.004 (3)
C10	0.082 (4)	0.069 (4)	0.040 (4)	0.011 (3)	0.020 (3)	−0.006 (3)
C11	0.060 (4)	0.053 (4)	0.038 (4)	0.003 (3)	0.002 (3)	0.002 (3)
C12	0.070 (4)	0.068 (4)	0.048 (4)	0.015 (3)	0.018 (3)	0.000 (3)
C13	0.052 (4)	0.054 (4)	0.045 (4)	0.004 (3)	0.009 (3)	0.006 (3)
Cl1	0.1068 (12)	0.0770 (11)	0.1405 (16)	0.0095 (10)	−0.0362 (12)	0.0368 (12)
N1	0.052 (3)	0.054 (3)	0.041 (3)	−0.001 (2)	0.009 (2)	−0.003 (2)
N2	0.063 (3)	0.042 (3)	0.039 (3)	−0.003 (2)	0.003 (2)	0.000 (2)
N3	0.063 (3)	0.053 (3)	0.059 (3)	−0.009 (2)	0.006 (3)	0.018 (3)
O1	0.043 (2)	0.059 (2)	0.046 (2)	0.0090 (17)	0.0044 (17)	0.0083 (18)
O2	0.116 (4)	0.135 (4)	0.068 (4)	−0.013 (3)	0.017 (3)	0.026 (3)

### Geometric parameters (Å, °)

**Table:**

Ni1—O1	2.054 (3)	C7—H7	0.9300
Ni1—O1^i^	2.054 (3)	C8—N1	1.296 (5)
Ni1—N2^i^	2.068 (4)	C8—H8	0.9300
Ni1—N2	2.068 (4)	C9—N1	1.474 (5)
Ni1—N1	2.102 (4)	C9—C10	1.519 (6)
Ni1—N1^i^	2.102 (4)	C9—H9A	0.9700
C1—O1	1.301 (5)	C9—H9B	0.9700
C1—C2	1.412 (6)	C10—C11	1.477 (6)
C1—C6	1.431 (6)	C10—H10A	0.9700
C2—C3	1.401 (6)	C10—H10B	0.9700
C2—C7	1.474 (7)	C11—C12	1.355 (6)
C3—C4	1.362 (7)	C11—N2	1.382 (5)
C3—H3	0.9300	C12—N3	1.356 (5)
C4—C5	1.376 (7)	C12—H12	0.9300
C4—Cl1	1.752 (5)	C13—N2	1.312 (5)
C5—C6	1.380 (6)	C13—N3	1.337 (5)
C5—H5	0.9300	C13—H13	0.9300
C6—C8	1.429 (6)	N3—H3A	0.8600
C7—O2	1.208 (5)		
			
O1—Ni1—O1^i^	87.37 (17)	C2—C7—H7	117.9
O1—Ni1—N2^i^	91.40 (14)	N1—C8—C6	128.9 (5)
O1^i^—Ni1—N2^i^	178.49 (14)	N1—C8—H8	115.5
O1—Ni1—N2	178.49 (14)	C6—C8—H8	115.5
O1^i^—Ni1—N2	91.40 (14)	N1—C9—C10	112.8 (4)
N2^i^—Ni1—N2	89.8 (2)	N1—C9—H9A	109.0
O1—Ni1—N1	88.84 (14)	C10—C9—H9A	109.0
O1^i^—Ni1—N1	89.72 (13)	N1—C9—H9B	109.0
N2^i^—Ni1—N1	91.14 (15)	C10—C9—H9B	109.0
N2—Ni1—N1	90.27 (15)	H9A—C9—H9B	107.8
O1—Ni1—N1^i^	89.72 (13)	C11—C10—C9	112.2 (4)
O1^i^—Ni1—N1^i^	88.84 (14)	C11—C10—H10A	109.2
N2^i^—Ni1—N1^i^	90.27 (15)	C9—C10—H10A	109.2
N2—Ni1—N1^i^	91.14 (15)	C11—C10—H10B	109.2
N1—Ni1—N1^i^	178.0 (2)	C9—C10—H10B	109.2
O1—C1—C2	120.9 (5)	H10A—C10—H10B	107.9
O1—C1—C6	122.8 (4)	C12—C11—N2	108.9 (5)
C2—C1—C6	116.2 (5)	C12—C11—C10	131.3 (5)
C3—C2—C1	122.2 (5)	N2—C11—C10	119.7 (5)
C3—C2—C7	117.6 (5)	C11—C12—N3	106.8 (5)
C1—C2—C7	120.2 (5)	C11—C12—H12	126.6
C4—C3—C2	119.4 (5)	N3—C12—H12	126.6
C4—C3—H3	120.3	N2—C13—N3	111.9 (5)
C2—C3—H3	120.3	N2—C13—H13	124.1
C3—C4—C5	120.3 (5)	N3—C13—H13	124.1
C3—C4—Cl1	120.3 (5)	C8—N1—C9	114.6 (4)
C5—C4—Cl1	119.4 (5)	C8—N1—Ni1	122.1 (3)
C4—C5—C6	121.9 (5)	C9—N1—Ni1	123.2 (3)
C4—C5—H5	119.0	C13—N2—C11	105.3 (4)
C6—C5—H5	119.0	C13—N2—Ni1	128.9 (4)
C5—C6—C8	115.6 (5)	C11—N2—Ni1	124.4 (3)
C5—C6—C1	119.9 (5)	C13—N3—C12	107.2 (4)
C8—C6—C1	124.4 (4)	C13—N3—H3A	126.4
O2—C7—C2	124.1 (6)	C12—N3—H3A	126.4
O2—C7—H7	117.9	C1—O1—Ni1	126.1 (3)
			
O1—C1—C2—C3	173.9 (4)	C10—C9—N1—Ni1	−28.0 (6)
C6—C1—C2—C3	−3.1 (6)	O1—Ni1—N1—C8	−17.2 (4)
O1—C1—C2—C7	−7.0 (7)	O1^i^—Ni1—N1—C8	−104.6 (4)
C6—C1—C2—C7	176.0 (4)	N2^i^—Ni1—N1—C8	74.1 (4)
C1—C2—C3—C4	1.5 (7)	N2—Ni1—N1—C8	164.0 (4)
C7—C2—C3—C4	−177.6 (5)	O1—Ni1—N1—C9	167.8 (4)
C2—C3—C4—C5	0.8 (8)	O1^i^—Ni1—N1—C9	80.4 (4)
C2—C3—C4—Cl1	−179.0 (3)	N2^i^—Ni1—N1—C9	−100.8 (4)
C3—C4—C5—C6	−1.3 (8)	N2—Ni1—N1—C9	−11.0 (4)
Cl1—C4—C5—C6	178.5 (4)	N3—C13—N2—C11	−0.2 (5)
C4—C5—C6—C8	−177.3 (5)	N3—C13—N2—Ni1	166.5 (3)
C4—C5—C6—C1	−0.4 (7)	C12—C11—N2—C13	0.6 (6)
O1—C1—C6—C5	−174.4 (4)	C10—C11—N2—C13	178.1 (5)
C2—C1—C6—C5	2.5 (6)	C12—C11—N2—Ni1	−166.8 (3)
O1—C1—C6—C8	2.2 (7)	C10—C11—N2—Ni1	10.7 (6)
C2—C1—C6—C8	179.2 (4)	N2^i^—Ni1—N2—C13	−52.1 (4)
C3—C2—C7—O2	−8.3 (8)	N1—Ni1—N2—C13	−143.3 (4)
C1—C2—C7—O2	172.5 (5)	N2^i^—Ni1—N2—C11	112.2 (4)
C5—C6—C8—N1	−173.5 (5)	N1—Ni1—N2—C11	21.0 (4)
C1—C6—C8—N1	9.8 (8)	N1^i^—Ni1—N2—C11	−157.6 (4)
N1—C9—C10—C11	69.3 (6)	N2—C13—N3—C12	−0.3 (6)
C9—C10—C11—C12	115.1 (6)	C11—C12—N3—C13	0.6 (6)
C9—C10—C11—N2	−61.8 (6)	C2—C1—O1—Ni1	157.3 (3)
N2—C11—C12—N3	−0.7 (6)	C6—C1—O1—Ni1	−25.9 (6)
C10—C11—C12—N3	−177.9 (5)	O1^i^—Ni1—O1—C1	118.8 (4)
C6—C8—N1—C9	178.7 (5)	N1—Ni1—O1—C1	29.0 (4)
C6—C8—N1—Ni1	3.3 (7)	N1^i^—Ni1—O1—C1	−152.4 (3)
C10—C9—N1—C8	156.6 (4)		

Symmetry codes: (i) *y*, *x*, −*z*.

### Hydrogen-bond geometry (Å, °)

**Table:**

*D*—H···*A*	*D*—H	H···*A*	*D*···*A*	*D*—H···*A*
C9—H9B···Cl1^ii^	0.97	2.82	3.475 (5)	125
C12—H12···O2^iii^	0.93	2.36	3.287 (7)	174
N3—H3A···O1^iv^	0.86	2.06	2.899 (5)	166

Symmetry codes: (ii) −*x*+1, −*y*, *z*−1/2; (iii) *y*+1/2, −*x*+1/2, *z*−3/4; (iv) −*y*+1/2, *x*+1/2, *z*−1/4.
